# Supplementary material for: Diagnostic Role and Prognostic Impact of PSAP Immunohistochemistry: A Tissue Microarray Study on 31,358 Cancer Tissues
Source: Diagnostics (Basel). 2023 Oct 18;13(20):3242. doi: 10.3390/diagnostics13203242 (PMC10606209; doi:10.3390/diagnostics13203242)
Supplement: Supplementary file 1 [file diagnostics-13-03242-s001.zip › Supplementary Figure S2.pdf]

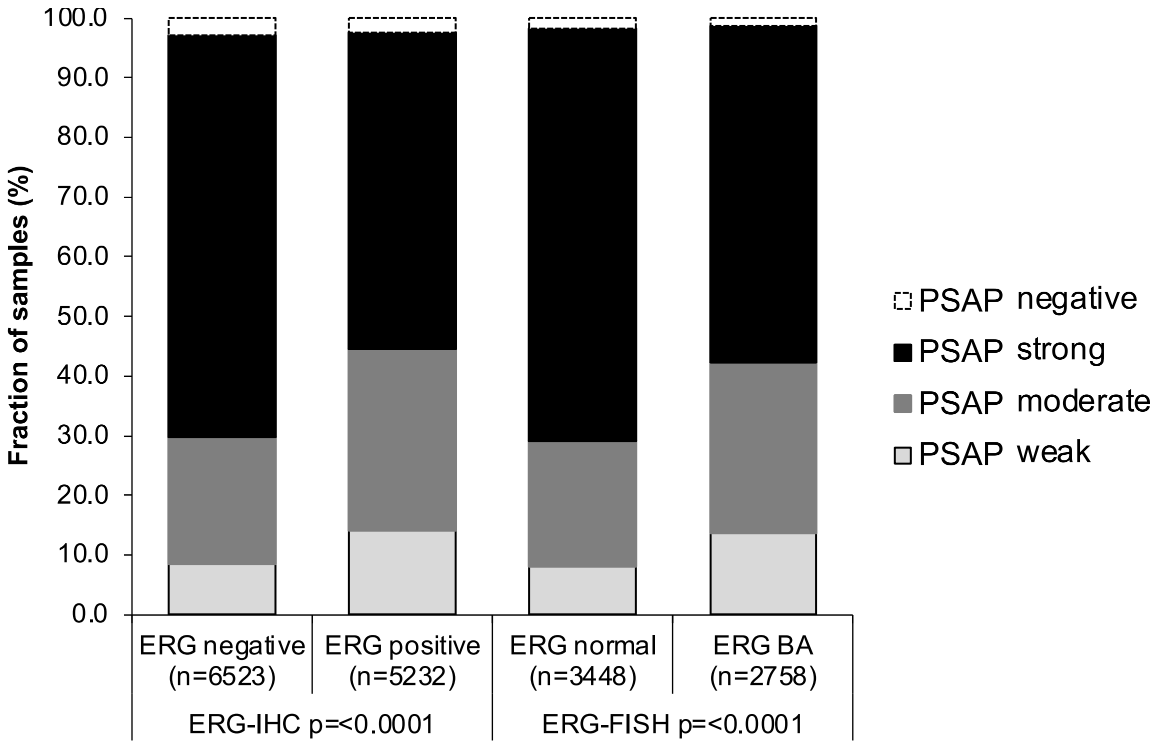
Fraction of samples (%)
100.0
90.0
80.0
70.0
60.0
50.0
40.0
30.0
20.0
10.0
0.0
ERG negative (n=6523)
ERG positive (n=5232)
ERG normal (n=3448)
ERG BA (n=2758)
ERG-IHC p=<0.0001
ERG-FISH p=<0.0001
PSAP negative
PSAP strong
PSAP moderate
PSAP weak
