# Supplementary material for: Diagnostic Role and Prognostic Impact of PSAP Immunohistochemistry: A Tissue Microarray Study on 31,358 Cancer Tissues
Source: Diagnostics (Basel). 2023 Oct 18;13(20):3242. doi: 10.3390/diagnostics13203242 (PMC10606209; doi:10.3390/diagnostics13203242)
Supplement: Supplementary file 1 [file diagnostics-13-03242-s001.zip › Table S1.pdf]

Table S1. Composition of the prostate cancer tissue microarray.

	No. of Patients (%)	
	Study Cohort on TMA<br><br>(n=17747)	Biochemical Relapse among Categories
**Follow-up (mo)**		
n	14464 (81.5%)	3612 (25%)
Mean	56.3	-
Median	48	-
**Age (y)**		
≤50	433 (2.4%)	66 (15.2%)
51-59	4341 (24.5%)	839 (19.3%)
60-69	9977 (56.4%)	2073 (20.8%)
≥70	2936 (16.6%)	634 (21.6%)
**Pretreatment PSA (ng/ml)**		
<4	2225 (12.6%)	313 (14.1%)
4-10	10520 (59.6%)	1696 (16.1%)
10-20	3662 (20.8%)	1043 (28.5%)
>20	1231 (7%)	545 (44.3%)
**pT stage (AJCC 2002)**		
pT2	11518 (65.2%)	1212 (10.5%)
pT3a	3842 (21.7%)	1121 (29.2%)
pT3b	2233 (12.6%)	1213 (54.3%)
pT4	85 (0.5%)	63 (74.1%)
**Gleason grade**		
≤3+3	3570 (20.3%)	264 (7.4%)
3+4	9336 (53%)	1436 (15.4%)
3+4 Tert.5	798 (4.5%)	165 (20.7%)
4+3	1733 (9.8%)	683 (39.4%)
4+3 Tert.5	1187 (6.7%)	487 (41%)
≥4+4	999 (5.7%)	531 (53.2%)
**pN stage**		
pN0	10636 (89.4%)	2243 (21.1%)
pN+	1255 (10.6%)	700 (55.8%)
**Surgical margin**		
Negative	14297 (80.8%)	2307 (16.1%)
Positive	3388 (19.2%)	1304 (38.5%)

NOTE: Percent in the column "Study cohort on TMA" refers to the fraction of samples across each category. Percent in colum "Biochemical relaps among categories" refers to the fraction of samples with biochemical relapse within each parameter in the different categories. Numbers do not always add up to 17,747 in the different categories because of cases with missing data. Abbreviation: AJCC, American Joint Committee on Cancer.
