# Supplementary material for: Diagnostic Role and Prognostic Impact of PSAP Immunohistochemistry: A Tissue Microarray Study on 31,358 Cancer Tissues
Source: Diagnostics (Basel). 2023 Oct 18;13(20):3242. doi: 10.3390/diagnostics13203242 (PMC10606209; doi:10.3390/diagnostics13203242)
Supplement: Supplementary file 1 [file diagnostics-13-03242-s001.zip › Table S2.pdf]

**Table S2.** PSAP and phenotype in ERG-negative prostate cancers.

| | n Evaluable | PSAP Immunostaining Result Negative (%) | Weak (%) | Moderate (%) | Strong (%) | p Value |
|---|---|---|---|---|---|---|
| **ERG negative cancers** | 6523 | 3.0 | 8.6 | 21.2 | 67.3 | |
| **Tumor stage** | | | | | | |
| pT2 | 4361 | 2.8 | 6.6 | 19.3 | 71.3 | |
| pT3a | 1289 | 2.5 | 9.7 | 22.8 | 65.0 | <0.0001 |
| pT3b-4 | 847 | 4.5 | 17.1 | 28.6 | 49.8 | |
| **Gleason score** | | | | | | |
| ≤3+3 | 1172 | 2.5 | 7.3 | 17.2 | 73.0 | |
| 3+4 | 3419 | 2.6 | 7.1 | 19.6 | 70.7 | |
| 3+4 Tert.5 | 328 | 2.7 | 7.6 | 22.9 | 66.8 | <0.0001 |
| 4+3 | 680 | 3.4 | 9.6 | 23.8 | 63.2 | |
| 4+3 Tert.5 | 455 | 4.0 | 12.1 | 30.3 | 53.6 | |
| ≥4+4 | 410 | 5.1 | 19.0 | 30.7 | 45.1 | |
| **quantitative Gleason score** | | | | | | |
| 3+4 ≤5% | 878 | 3.6 | 7.5 | 17.3 | 71.5 | |
| 3+4 6-10% | 854 | 1.8 | 6.0 | 19.1 | 73.2 | |
| 3+4 11-20% | 726 | 2.1 | 6.7 | 19.4 | 71.8 | |
| 3+4 21-30% | 378 | 2.6 | 8.2 | 20.9 | 68.3 | <0.0001 |
| 3+4 31-49% | 336 | 2.4 | 8.9 | 23.8 | 64.9 | |
| 4+3 50-60% | 267 | 2.7 | 7.6 | 22.9 | 66.8 | |
| 4+3 61-80% | 245 | 1.9 | 8.2 | 22.5 | 67.4 | |
| 4+3 >80% | 72 | 2.9 | 11.4 | 25.3 | 60.4 | |
| **Lymph node metastasis** | | | | | | |
| N0 | 3979 | 2.9 | 8.7 | 22.0 | 66.4 | <0.0001 |
| N+ | 449 | 5.6 | 18.3 | 30.1 | 46.1 | |
| **Preoperative PSA level (ng/ml)** | | | | | | |
| <4 | 666 | 4.1 | 12.6 | 21.5 | 61.9 | |
| 4-10 | 540 | 3.5 | 11.7 | 27.6 | 57.2 | <0.0001 |
| 10-20 | 3764 | 2.8 | 7.3 | 19.9 | 70.0 | |
| >20 | 1520 | 2.8 | 8.7 | 22.4 | 66.2 | |
| **Surgical margin** | | | | | | |
| negative | 5206 | 3.2 | 7.6 | 20.6 | 68.6 | <0.0001 |
| positive | 1295 | 2.2 | 12.4 | 24.0 | 61.5 | |
