# Supplementary material for: Diagnostic Role and Prognostic Impact of PSAP Immunohistochemistry: A Tissue Microarray Study on 31,358 Cancer Tissues
Source: Diagnostics (Basel). 2023 Oct 18;13(20):3242. doi: 10.3390/diagnostics13203242 (PMC10606209; doi:10.3390/diagnostics13203242)
Supplement: Supplementary file 1 [file diagnostics-13-03242-s001.zip › Table S3.pdf]

Table S3. PSAP immunostaining and phenotype in ERG-positive prostate cancers.

	n Evaluable	PSAP Immunostaining Result				p Value
		Negative (%)	Weak (%)	Moderate (%)	Strong (%)	
**ERG positive cancers**	5232	2.5	14.0	30.5	53.0	
**Tumor stage**						
pT2	2993	2.5	12.1	30.0	55.4	<0.0001
pT3a	1412	2.1	14.5	31.2	52.2	
pT3b-4	801	3.0	20.3	30.7	45.9	
**Gleason score**						
≤3+3	964	2.7	10.6	27.7	59.0	<0.0001
3+4	2843	1.9	13.4	30.8	53.9	
3+4 Tert.5	205	2.4	14.6	35.6	47.3	
4+3	528	3.2	16.1	28.2	52.5	
4+3 Tert.5	363	2.2	20.4	34.2	43.3	
≥4+4	262	5.0	20.6	32.8	41.6	
**quantitative Gleason score**						
3+4 ≤5%	675	2.4	12.6	31.1	53.9	<0.0001
3+4 6-10%	695	2.4	11.8	30.6	55.1	
3+4 11-20%	618	1.3	13.4	29.9	55.3	
3+4 21-30%	331	0.9	17.5	27.8	53.8	
3+4 31-49%	262	0.8	15.6	31.3	52.3	
4+3 50-60%	219	2.4	14.6	35.6	47.3	
4+3 61-80%	184	2.3	15.1	24.7	58.0	
4+3 >80%	31	4.9	15.8	29.3	50.0	
**Lymph node metastasis**						
N0	3049	2.4	14.2	32.1	51.3	0.0082
N+	462	3.0	20.1	30.7	46.1	
**Preoperative PSA level (ng/ml)**						
<4	683	4.0	15.5	29.7	50.8	0.0047
4-10	352	3.1	19.9	28.1	48.9	
10-20	3138	2.3	12.8	30.9	54.0	
>20	1015	1.8	14.5	30.1	53.6	
**Surgical margin**						
negative	4044	2.5	13.5	30.5	53.5	0.2732
positive	1163	2.5	15.7	30.4	51.3	
