# Supplementary material for: Diagnostic Role and Prognostic Impact of PSAP Immunohistochemistry: A Tissue Microarray Study on 31,358 Cancer Tissues
Source: Diagnostics (Basel). 2023 Oct 18;13(20):3242. doi: 10.3390/diagnostics13203242 (PMC10606209; doi:10.3390/diagnostics13203242)
Supplement: Supplementary file 1 [file diagnostics-13-03242-s001.zip › Table S4.pdf]

Table S4. Multivariate analyses.

Tumor Subset	Scenario	n Analyzable	p -Value							
			Preoperative PSA-Level	pT Stage	cT Stage	Gleason Score Prostatectomy	Gleason Score Biopsy	pN Stage	R Status	PSAP-Expression
all cancers	1	7942	<0.0001	<0.0001	-	<0.0001	-	<0.0001	0.0002	0.5301
	2	12285	<0.0001	<0.0001	-	<0.0001	-	-	<0.0001	0.2073
	3	12087	<0.0001	-	<0.0001	<0.0001	-	-	-	0.0293
	4	10121	<0.0001	-	<0.0001	-	<0.0001	-	-	<0.0001
ERG negative cancers	1	3376	<0.0001	<0.0001	-	<0.0001	-	<0.0001	0.5348	0.4052
	2	5165	<0.0001	<0.0001	-	<0.0001	-	-	0.0062	0.0051
	3	5089	<0.0001	-	<0.0001	<0.0001	-	-	-	<0.0001
	4	4230	<0.0001	-	<0.0001	-	<0.0001	-	-	<0.0001
ERG postive cancers	1	2720	<0.0001	<0.0001	-	<0.0001	-	<0.0001	0.0003	0.6072
	2	4198	<0.0001	<0.0001	-	<0.0001	-	-	<0.0001	0.2878
	3	4113	<0.0001	-	<0.0001	<0.0001	-	-	-	0.2740
	4	3497	<0.0001	-	<0.0001	-	<0.0001	-	-	0.1414

Abbreviations: pT: pathological tumor stage, pN: pathological lymph node status, R: resction margin
