# Supplementary figures and images for: Diagnostic Role and Prognostic Impact of PSAP Immunohistochemistry: A Tissue Microarray Study on 31,358 Cancer Tissues
Source: Diagnostics (Basel). 2023 Oct 18;13(20):3242. doi: 10.3390/diagnostics13203242 (PMC10606209; doi:10.3390/diagnostics13203242)

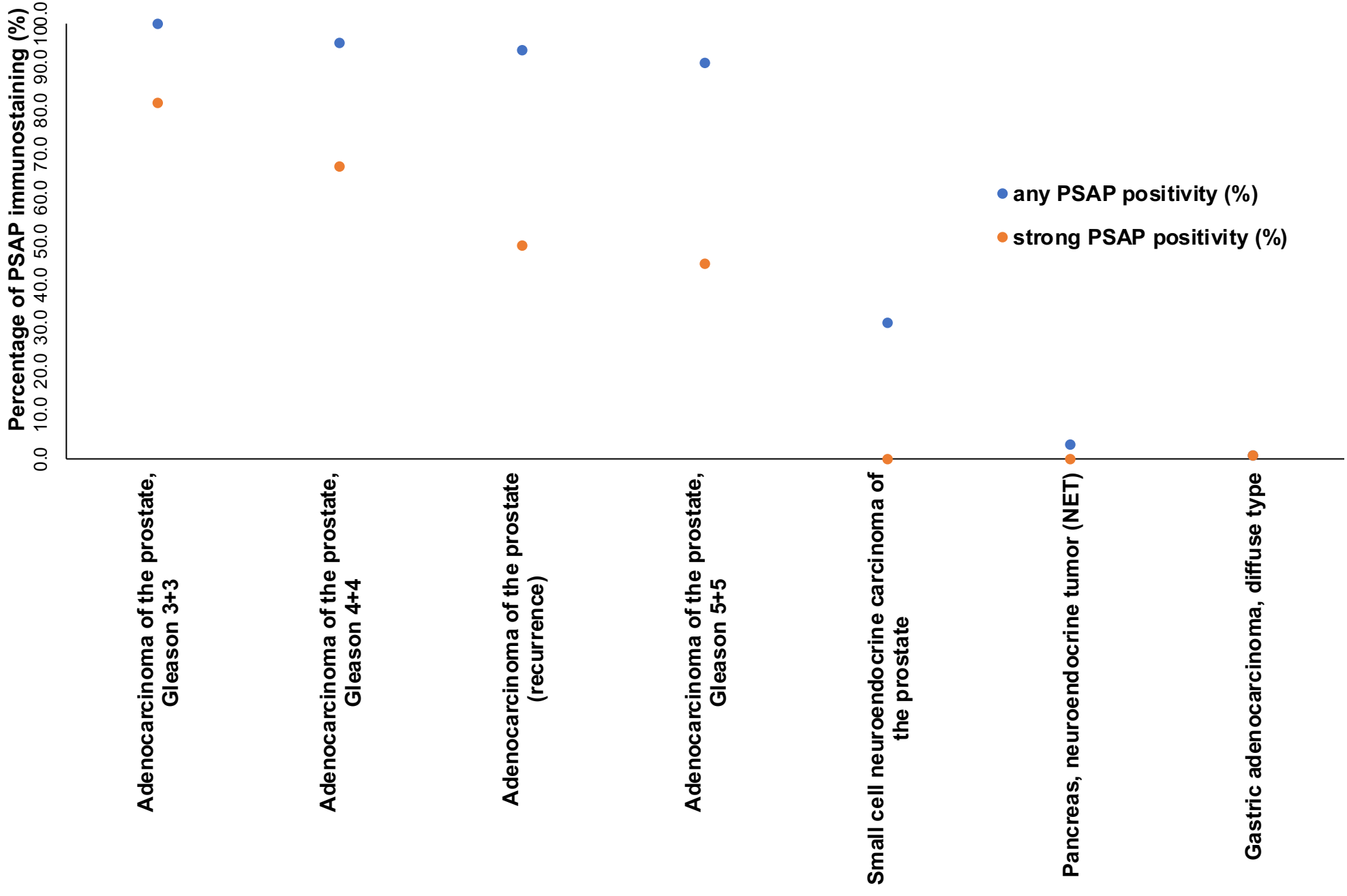

Supplement: Supplementary file 1 [file diagnostics-13-03242-s001.zip › Supplementary Figure S1.pdf]

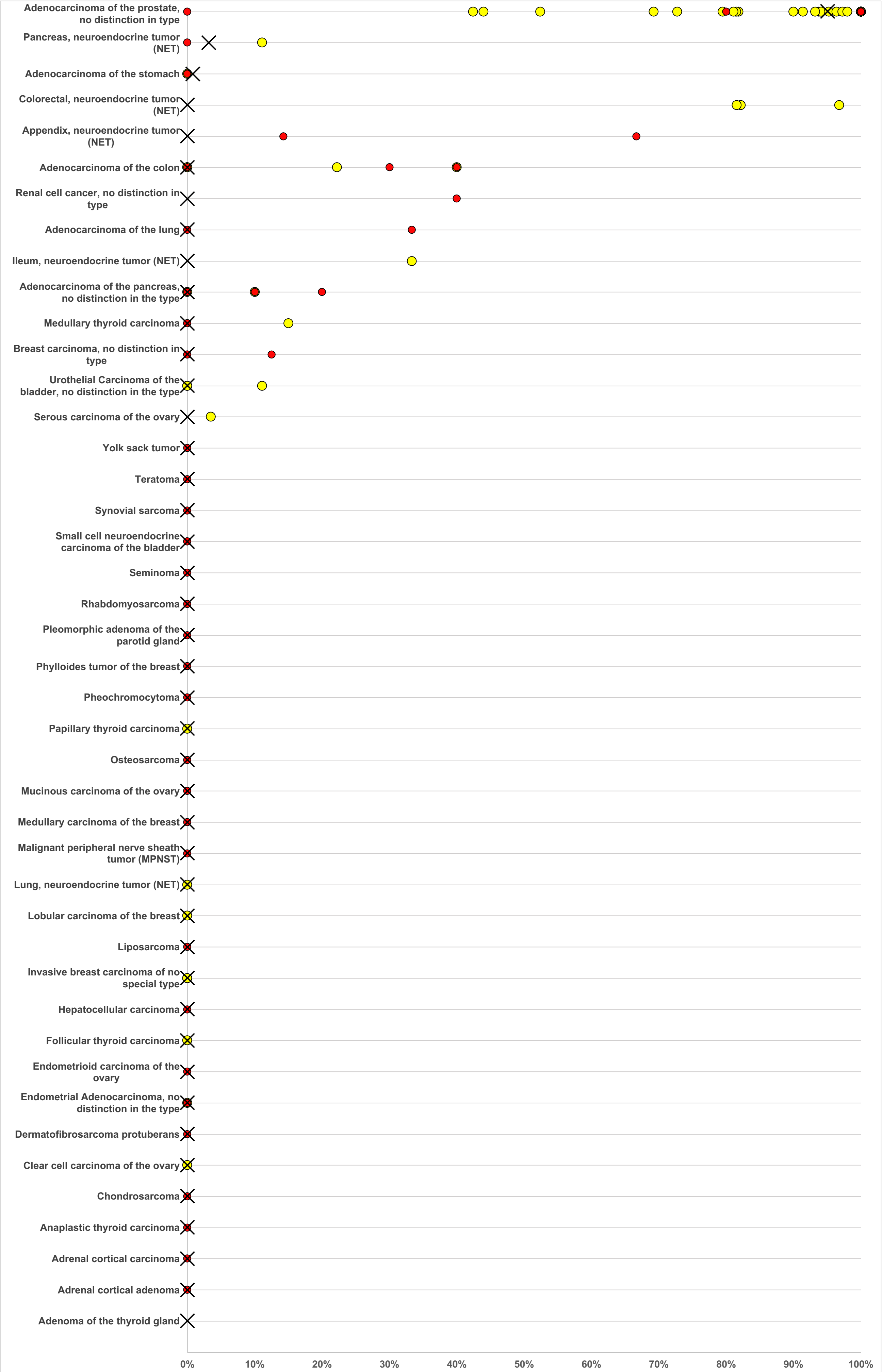

Supplement: Supplementary file 1 [file diagnostics-13-03242-s001.zip › Supplementary Figure S3.pdf]
